# Neurodevelopmental continuum and pathogenic CNV detection in adult onset psychiatric disorders: microarray analysis in psychiatric clinical practice

**DOI:** 10.1192/j.eurpsy.2022.1748

**Published:** 2022-09-01

**Authors:** Á. Ruiz De Pellón Santamaría

**Affiliations:** Donostia University Hospital, Psychiatry, Donostia, Spain

**Keywords:** schizophrénia, CNVs, Neurodevelopmental disorders, microarray analysis

## Abstract

**Introduction:**

Structural variations of DNA, such as copy number variations (CNVs), are important contributors to risk for human diseases. Several CNVs have been associated with an increased risk of early-onset neurodevelopmental disorders (NDD), adult-onset psychiatry disorders and physical comorbidities. While in Pediatrics the microarray is the first-line genetic analysis technique in the study of child onset NDD, its use in psychiatry care of young/adult onset NDD has been limited to research purposes.

**Objectives:**

Review of the diagnostic yield of the use of microarrays analysis in psychiatric clinical practice of severe mental disorder care in adults, according to the concept of a neurodevelopmental continuum.

**Methods:**

An exploratory literature review on the topic in PubMed, including the terms: “copy number variants/CNVs” AND “neurodevelopmental delay/disorders, congenital anomalies/malformations, ADHD, autism/ASD, learning disabilities, epilepsy, Tourette, schizophrenia, bipolar, behaviour”.

**Results:**

The prevalence of carriers of pathogenic or likely pathogenic CNVs among the different NDD phenotypes investigated by microarray analysis ranged from 3-22.5%. The majority of studies in adult psychiatric populations examined schizophrenia. Intellectual disability, autism spectrum disorders, dysmorphic features and multiple NDD/psychiatric diagnoses were described as predictors of an increased diagnostic yield of microarray testing.

**Conclusions:**

While CNV testing is frequent in early-onset NDD; microarray analysis has not been established in psychiatric clinical practice despite the evidence of a high prevalence of findings in adult-onset NDD. The potential benefits in the detection of CNV are associated with physical comorbidities detection, understanding of pathogenesis of disease or genetic counseling. High-quality research designs are required before a routine clinical use.

**Disclosure:**

No significant relationships.

